# The Southern European Atlantic diet and depression risk: a European multicohort study

**DOI:** 10.1038/s41380-023-02125-9

**Published:** 2023-06-23

**Authors:** Adrián Carballo-Casla, Denes Stefler, Rosario Ortolá, Yuntao Chen, Anika Knuppel, Milagros Ruiz, Magdalena Kozela, Ruzena Kubinova, Andrzej Pajak, Fernando Rodríguez-Artalejo, Eric J. Brunner, Martin Bobak

**Affiliations:** 1 Department of Preventive Medicine and Public Health, Universidad Autónoma de Madrid, Madrid, Spain. Center for Networked Biomedical Research in Epidemiology and Public Health (CIBERESP), Madrid, Spain; 2 Department of Epidemiology and Public Health, University College London, London, UK; 3 Aging Research Center, Department of Neurobiology, Care Sciences and Society, Karolinska Institutet & Stockholm University, Stockholm, Sweden; 4 School of Health and Social Care, University of Essex, Colchester, UK; 5 Department of Epidemiology and Population Studies, Jagiellonian University Medical College, Krakow, Poland; 6 National Institute of Public Health, Prague, Czech Republic; 7 IMDEA Research Institute on Food & Health Sciences. CEI UAM+CSIC, Madrid, Spain

**Keywords:** Depression, Psychology

## Abstract

The Southern European Atlantic diet (SEAD) is the traditional dietary pattern of north-western Spain and northern Portugal, but it may resemble that of other European countries. The SEAD has been found associated with lower risk for myocardial infarction and mortality. Since dietary patterns may also influence mental health, we examined the association between the SEAD and depression risk in southern, central, eastern, and western European populations. We conducted a prospective analysis of five cohorts (13,297 participants aged 45–92 years, free of depression at baseline): Seniors-ENRICA-1 and Seniors-ENRICA-2 (Spain), HAPIEE (Czechia and Poland), and Whitehall-II (United Kingdom). The SEAD comprised cod, other fresh fish, red meat and pork products, dairy, legumes and vegetables, vegetable soup, potatoes, whole-grain bread, and moderate wine consumption. Depression at follow-up was defined according to presence of depressive symptoms (based on available scales), use of prescribed antidepressants, inpatient admissions, or self-reported diagnosis. Associations were adjusted for sociodemographic, lifestyle, and dietary variables. During a median follow-up of 3.9 years (interquartile range 3.4–4.9), there were 1437 new depression cases. Higher adherence to the SEAD was associated with lower depression risk in the pooled sample. Individual food groups showed a similar tendency, albeit non-significant. The fully adjusted odds ratio (95% confidence interval) per 1-standard deviation increment in the SEAD was 0.91 (0.86, 0.96). This association was rather consistent across countries [Spain = 0.86 (0.75, 0.99), Czechia = 0.86 (0.75, 0.99), Poland = 0.97 (0.89, 1.06), United Kingdom = 0.85 (0.75, 0.97); *p* for interaction = 0.24], and was of similar magnitude as that found for existing healthy dietary patterns. In conclusion, the SEAD was associated with lower depression risk across European populations. This may support the development of mood disorder guidelines for Southern European Atlantic regions based on their traditional diet, and for central, eastern, and western European populations based on the SEAD food groups that are culturally rooted in these places.

## Introduction

The Southern European Atlantic diet (SEAD) is the traditional dietary pattern of north-western Spain and northern Portugal. In these regions, staple foods were fish -particularly cod-, red meat and pork products, dairy, legumes, vegetables and potatoes -often eaten in soups-, whole-grain bread, and wine [1–4] Conversely, the consumption of olive oil, fresh fruits, and nuts was not widespread until the second half of the twentieth century [1–3]. Though such a combination of food groups is distinctive to the SEAD, several of these foods are shared with traditional diets of Nordic, central, eastern, and western European countries [5, 6].

The consumption of some of the SEAD food groups is partially inconsistent with current healthy dietary recommendations. Unlike the Alternate Healthy Eating Index (AHEI) or the Mediterranean Diet Score (MDS) [7, 8], the SEAD includes red meat, pork products, and potatoes, which could be linked to adverse health outcomes [9–12]. Still, the healthier food components of SEAD may predominate over the less healthy ones, as increased adherence to the SEAD has been associated with healthier gut microbiota, reduced levels of several cardiovascular risk factors (C-reactive protein, triglycerides, insulin, insulin resistance, pulse wave velocity, systolic blood pressure, total cholesterol, body mass index [BMI], and waist circumference), and decreased risk of myocardial infarction and all-cause mortality [3, 13–21].

Nevertheless, existing healthy dietary patterns may also play a favourable role in mental health conditions [22], while associations with the SEAD are uncertain. Although consumption of several SEAD food groups (e.g., vegetables, fish, light-to-moderate wine consumption) has been linked to lower depression risk [22, 23], prevalence of depressive disorder may be higher in Spain and Portugal than in other European countries [24, 25]. Importantly, most -if not all- studies on the SEAD have been conducted in its countries of origin, so the external validity of their findings would be unclear if not reproduced beyond their borders.

To assess the impact of the SEAD on of mood disorders, and its generalizability from Southern European Atlantic countries to other European populations, we examined the association between adherence to the SEAD and depression risk in five cohorts from four European countries (Spain, Czechia, Poland, and the United Kingdom). We also examined the contribution of individual SEAD food groups to this association.

## Patients and methods

### Study design and participants

Five cohort studies were included in the analyses. The “Impact of Dietary Patterns and Sedentary Behaviour on the Accumulation of Health Deficits and Physical Resilience in Older Adults” (Seniors-ENRICA-1 and Seniors-ENRICA-2) are two cohorts of Spanish community-dwelling individuals aged ≥60 years and ≥65 years, respectively. We took data from the first and second follow-up waves of Seniors-ENRICA-1 (2012 and 2014-2015, respectively), and from the baseline and first follow-up waves of Seniors-ENRICA-2 (2017 and 2019, respectively), as that was when information on depressive symptoms was first collected. Data on sociodemographic variables, lifestyle, and morbidity were gathered through telephone interviews, whereas detailed diet histories, comprehensive sets of physical measurements, and blood tests were collected at home visits by trained personnel. The Clinical Research Ethics Committee of the “La Paz” University Hospital in Madrid approved the research protocols, and all subjects gave written informed consent [26, 27].

The “Health, Alcohol and Psychosocial factors In Eastern Europe” (HAPIEE) cohort, set up in 2002–2005, recruited random samples of men and women aged 45–69 years from six cities in Czechia and Krakow (Poland). Data collection consisted of an interview that gathered data on health (including depressive symptoms), lifestyle, diet via a food frequency questionnaire (FFQ), and socioeconomic circumstances. A short examination, including physical measurements and a blood test, was also conducted. A second data collection wave followed in 2006–2008. The study was approved by the ethics committee at University College London and by the ethics committee in each participating centre. All participants gave written informed consent [28].

The Whitehall-II is a cohort study of civil servants from 20 civil service departments in London (United Kingdom). Participants undergo medical examinations and fill out an FFQ every 5 years, and they complete an array of questionnaires in and between these screening phases [29–31]. We used data from the seventh and ninth phases of the study, which took place in 2002–2004 and 2007–2009, respectively (note that these were the first waves that collected information on depressive symptoms via dedicated scales). The University College London Ethics Committee approved the study. After the subjects were given a complete description of the study, written informed consent was obtained from all participants [32].

### Study variables

#### Diet

In the Seniors-ENRICA cohorts, food consumption was obtained with a validated electronic diet history. Subjects could report up to 861 foods and recipes habitually consumed in the country. Portion sizes were estimated with the help of 127 digitized photographs and household measures. Nutrient and energy intake were derived from Spanish and other standard food composition tables [33]. In HAPIEE and Whitehall-II, dietary data were collected with a semi-quantitative FFQ that consisted of 136, 147, and 116 foods and beverages in Czechia, Poland, and the United Kingdom, respectively -note that the FFQ used in HAPIEE was an expanded version of that used in Whitehall-II. In these questionnaires, participants indicated how frequently they consumed foods and drinks by using a 9-point scale, ranging from “never, or less than once a month” to “more than 6-times a day”. The McCance and Widdowson’s food composition tables were used to calculate nutrient and energy intake [29, 34].

To assess the adherence to the SEAD, we used a scoring method proposed by Oliveira et al., which includes the following nine food groups: fresh fish -excluding cod-, cod, red meat and pork products, dairy, legumes and vegetables -excluding those consumed in soup-, vegetable soup, potatoes -regardless of the cooking method-, whole-grain bread, and wine [3]. The rationale for using this scoring method was twofold: firstly, to maximize the comparability of our findings, as it has been used by most studies on the SEAD [3, 13–18, 21]; secondly, to allow for data harmonization, as the consumption of these nine food groups was collected in all the participating cohorts [29, 33, 34].

We computed the consumption of every food group of the SEAD -except wine- as grams/1000 kilocalories/day, and calculated its sex-specific median. The subjects who were above the median consumption scored 1 point, whereas those who were at or below it scored 0 points. Regarding wine consumption, men who drank >0 and ≤2 glasses/day and women who drank >0 and ≤1 glasses/day were given 1 point, whereas no points were given for >2 glasses/day in men, >1 glass/day in women or 0 glasses/day. The adherence to the SEAD was computed as the sum of scores of these nine food groups; it ranged from 0 to 9, with higher values indicating better adherence [3].

To put the SEAD in context, we compared the study associations with those of two dietary patterns that may lower the odds of depressive outcomes [22]: the Alternate Healthy Eating Index, whose food groups were selected based on its association with chronic disease risk [7], and the Mediterranean Diet Score, which reflects adherence to the traditional Mediterranean diet [8].

#### Depression

In the Seniors-ENRICA cohorts, depression at baseline and follow-up was defined as a score ≥3 on the 10-item version of the Geriatric Depression Scale (GDS-10), use of prescribed antidepressant medication (checked by study staff against drug packages at home), or self-reported medical diagnosis (i.e., a positive answer to the question “did a physician tell you if you currently have, or had in the past year, depression -requiring treatment-?”) [26, 35].

A score ≥16 on the 20-item version of the Centre for Epidemiological Studies Depression scale (CESD-20) was used to define depression at baseline in HAPIEE. Depressive symptoms at follow-up were assessed using the 10-item version of the Centre for Epidemiological Studies Depression scale (CESD-10). A score ≥4 was used to define depression caseness [28, 36].

In Whitehall-II, depression at baseline and follow-up was defined as a score ≥16 on the CESD-20, reported use of prescribed antidepressant medication, or primary/secondary diagnoses of depression or major depressive episode in that year or the previous year (taken from the national hospital episode statistics database of inpatient data) [32, 37].

The depression scores were only computed for the participants with sufficient data (i.e., those who responded to at least 8 items of the GDS-10/CESD-10, or at least 16 items of the CESD-20) [36].

#### Covariates

We used baseline data on several potential confounders of the association between the SEAD and depression risk. Firstly, sociodemographic variables, specifically sex, age, educational level (primary or less, secondary or vocational, university, or no data), marital status (single, married/cohabiting, divorced/separated or widowed, or no data), and material deprivation (i.e., how often individuals had difficulties with paying bills, or did not have enough money for food, clothing, and housing-related expenses). Material deprivation was categorized into lower, intermediate, or higher (roughly evenly spaced categories), or no data. Secondly, we used data on lifestyle-related variables: measured BMI (<25, 25–30, ≥30 kg/m^2^, or no data), self-reported tobacco smoking (never, former, current, or no data), and self-reported leisure-time physical activity (hours/week). Finally, to account for possible dietary confounding of the study associations, we computed the consumption of common food groups not included in the SEAD (fruits, nuts, and sugar-sweetened beverages) [26–28, 32].

### Statistical analyses

#### Analytical sample

Of the 32,344 subjects recruited at baseline (2519 from the wave 1 of Seniors-ENRICA-1, 3273 from Seniors-ENRICA-2, 19,585 from HAPIEE, and 6967 from the phase 7 of Whitehall-II), 11,929 were deemed ineligible to minimize the potential for reverse causation (7149 had depression and 6800 had cardiovascular disease or cancer history at baseline; note that some subjects suffered from multiple conditions). From these 20,415 participants, we excluded 7118 subjects with inadequate data (1501 had no information on diet and 6194 on depression; note that some subjects lacked data in both variables). Hence, the pooled analytical sample comprised 13,297 individuals (Supplementary Fig. 1).

#### Main statistical analyses

The association between the SEAD and depression risk was summarized with odds ratios (OR) and their two-sided 95% confidence interval (CI), estimated with logistic regression models, as data on depression was collected at only two time points, and not continuously. Analyses were conducted in the pooled sample. Pooled estimates were adjusted for country, whereas country-specific estimates were obtained from models with interaction terms between the SEAD and each country, using the *lincom* command in Stata^®^ (StataCorp LLC), version 17.0. To control for potential confounding, three incrementally adjusted models were used: (1) adjusted for sociodemographic characteristics, (2) additionally adjusted for lifestyle variables, and (3) further adjusted for common food groups not included in the SEAD. To prevent ill health from influencing food consumption, analyses excluded the subjects with depression, cardiovascular disease history, or cancer history at baseline, as mentioned in the previous paragraph [38]. The medical diagnoses of the latter two conditions were self-reported in the Seniors-ENRICA and HAPIEE cohorts, while they were verified through primary care and hospital records in the Whitehall-II study [26–28, 32]. Note that the use of cardiovascular medication (e.g., lipid-lowering, glucose-lowering, or antihypertensives) in the absence of a medical diagnosis of coronary heart disease, stroke, or heart failure did not constitute an exclusion criterion.

The country-specific adherence to the SEAD was modelled as (1) a continuous variable [per 1-standard deviation (SD) increment]; (2) a trichotomous variable (calculated roughly as the lowest, intermediate and highest tertiles), using the lowest one (reflecting the least adherence) as reference; and (3) a restricted cubic spline (knots located at the 10th, 50th, and 90th percentiles (Supplementary Appendix 1) [39]. The adherence to the AHEI and the MDS was modelled alike. When examining the SEAD food groups, they were entered in the models as dichotomous variables (above or below the aforementioned food- and sex-specific consumption thresholds). To assure that the latter associations were independent of the consumption of other foods, model 3 was here adjusted for all SEAD food groups -except for the one being examined.

#### Sensitivity analyses and interactions

We conducted several sensitivity analyses: First, we calculated an alternate version of the SEAD, optimized for potential public health interventions. Here, we reversely scored the consumption of red meat/pork products and potatoes and did not score wine consumption. To avoid potential confounding, these analyses were additionally adjusted for wine consumption. Second, since beer and other alcoholic beverages may be consumed more often than wine in some central, eastern, and western European countries, we computed the SEAD considering total alcohol intake instead of wine consumption. Specifically, men who had >0 and ≤20 g/day of alcohol and women who had >0 and ≤10 g/day were given 1 point, whereas no points were given for >20 g/day in men, >10 g/day in women, or 0 g/day. Third, since BMI may mediate -rather than confound- the association between the SEAD and depression risk, we did not adjust the analyses for this variable. Fourth, to further minimize the potential for reverse causation, we removed the subjects with diabetes (blood glucose levels ≥7 mmol/L, treated with antidiabetic drugs, or self-reported diagnosis) or chronic lung disease (self-reported diagnosis) at baseline.

Lastly, we conducted two additional analyses to address the issue of subthreshold depression. On one hand, an increased number of depression symptoms at baseline could have been predictive of depression at follow-up. Accordingly, we adjusted for baseline risk of probable depression, categorized as follows: (1) Lower (no depression symptoms at baseline); (2) Intermediate (score = 1 on the GDS-10 or scores 1–7 on the CESD-20); (3) Higher (score = 2 on the GDS-10 or scores 8–15 on the CESD-20); or (4) No data. On the other hand, some depression cases at baseline and follow-up -particularly in their milder forms- were possibly missed, because the sensitivity of the depression scales and cut-offs is not perfect [35, 36]. To overcome this potential misclassification, we used fewer depressive symptoms to define depression at baseline and follow-up (score ≥2 on the GDS-10, score ≥3 on the CESD-10, or score ≥12 on the CESD-20).

We also examined if the sociodemographic, lifestyle, and dietary variables included in the models modified the main study associations by using likelihood-ratio tests that compared models with and without interaction terms, defined as the product of the SEAD by said variables.

## Results

### Description of study participants

Compared to individuals with the lowest adherence to the SEAD (Supplementary Appendix 1), those with the highest adherence were older and more often men, married or cohabiting. They had a higher educational level and lower material deprivation. Their BMI was higher as well, though they were less likely to smoke. Finally, their consumption of fruits, nuts, and sugar-sweetened beverages was lower (Table 1). The distribution of characteristics of study participants across countries is shown in Supplementary Table 1.

**Table 1:** Characteristics of the pooled sample, by adherence to the Southern European Atlantic diet.

	Southern European Atlantic diet			
	Lowest	Intermediate	Highest	Pooled
*n*	3510	6217	3570	13,297
Sex-Male (%)	1956 (55.7)	3557 (57.2)	2140 (59.9)*	7653 (57.6)
Age (years)	60.0 (8.47)	61.4 (8.48)	61.6 (7.96)*	61.1 (8.36)
Educational level (%)				
Primary or less	523 (14.9)	958 (15.4)	432 (12.1)*	1913 (14.4)
Secondary or vocational	2023 (57.6)	3442 (55.4)	1981 (55.5)	7446 (56.0)
University	943 (26.9)	1768 (28.4)	1119 (31.3)	3830 (28.8)
No data	21 (0.60)	49 (0.79)	38 (1.06)	108 (0.81)
Marital status (%)				
Single	285 (8.12)	386 (6.21)	208 (5.83)*	879 (6.61)
Married/cohabiting	2622 (74.7)	4840 (77.9)	2890 (81.0)	10,352 (77.9)
Divorced/separated or widowed	597 (17.0)	973 (15.7)	460 (12.9)	2030 (15.3)
No data	6 (0.17)	18 (0.29)	12 (0.34)	36 (0.27)
Material deprivation (%)				
Lower	1934 (55.1)	3516 (56.6)	2118 (59.3)*	7568 (56.9)
Intermediate	1372 (39.1)	2427 (39.0)	1336 (37.4)	5135 (38.6)
Higher	173 (4.93)	220 (3.54)	81 (2.27)	474 (3.56)
No data	31 (0.88)	54 (0.87)	35 (0.98)	120 (0.90)
Tobacco smoking (%)				
Never	1602 (45.6)	2971 (47.8)	1705 (47.8)*	6278 (47.2)
Former	1099 (31.3)	2122 (34.1)	1321 (37.0)	4542 (34.2)
Current	785 (22.4)	1102 (17.7)	532 (14.9)	2419 (18.2)
No data	24 (0.68)	22 (0.35)	12 (0.34)	58 (0.44)
Body mass index (%)				
<25 kg/m^2^	1149 (32.7)	1872 (30.1)	990 (27.7)*	4011 (30.2)
25–30 kg/m^2^	1576 (44.9)	2883 (46.4)	1683 (47.1)	6142 (46.2)
≥30 kg/m^2^	783 (22.3)	1453 (23.4)	884 (24.8)	3120 (23.5)
No data	2 (0.06)	9 (0.14)	13 (0.36)	24 (0.18)
Physical activity (hours/week)	15.4 (11.9)	15.5 (11.7)	15.7 (11.2)	15.5 (11.6)
Fruits (g/day)	377 (429)	351 (293)	334 (217)*	353 (319)
Nuts (g/day)	6.58 (15.4)	5.73 (11.8)	4.39 (8.58)*	5.60 (12.2)
Sugar-sweetened beverages (g/day)	181 (254)	140 (203)	113 (154)*	143 (208)

Values are numbers (%) or means (standard deviations).

*P value < 0.05 (two-sided) for differences in means (ANOVA) or proportions (Pearson’s chi-squared) across categories of adherence to the Southern European Atlantic diet.

The SEAD food groups, ranked from highest to lowest consumption, were dairy, legumes and vegetables, potatoes, red meat and pork products, vegetable soup, whole-grain bread, fresh fish (excluding cod), and cod. Most participants did not drink wine or had <1 glass/day (Supplementary Table 2).

### Main results

**Fig. 1:**
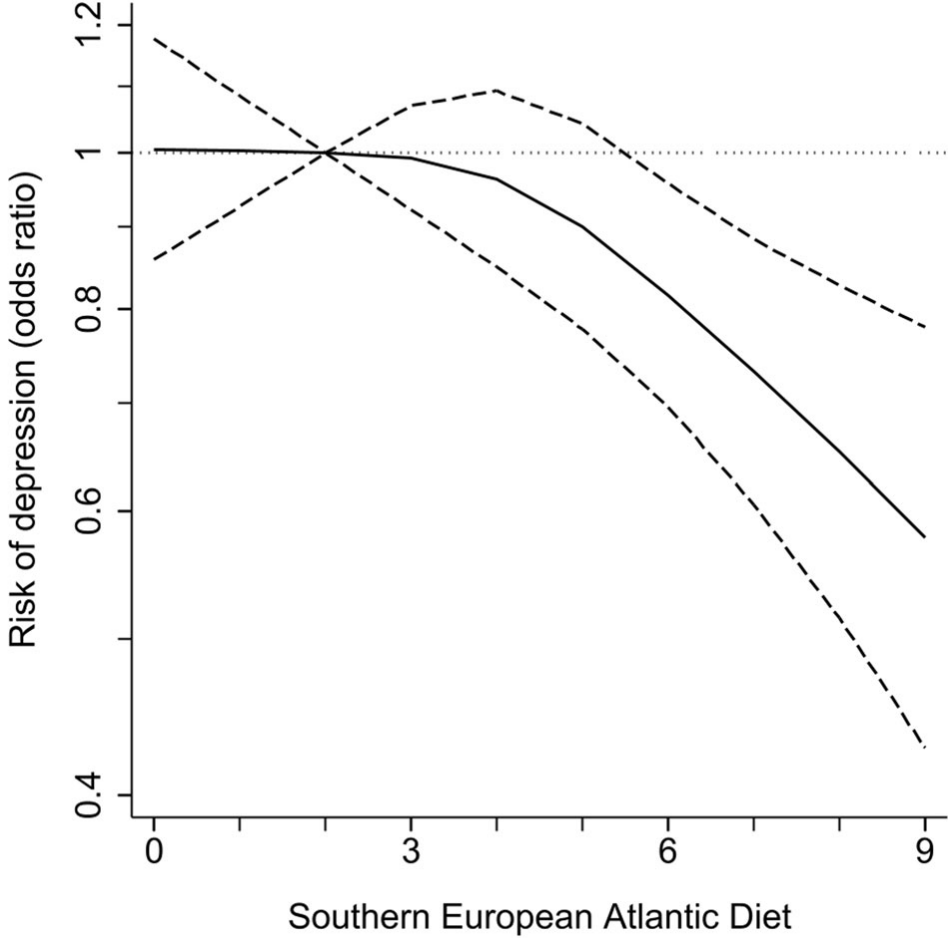
Odds ratios (two-sided 95% confidence interval) for the association between adherence to the Southern European Atlantic diet and 3.9-year risk of depression in the pooled sample. Logistic regression model adjusted as Model 3 in Table 2: country, sex, age, educational level (primary or less, secondary, university, or no data), marital status (single, married/cohabiting, divorced/separated/widowed, or no data), material deprivation (lower, intermediate, higher, or no data), smoking status (never, former, current, or no data), leisure-time physical activity (hours/week), body mass index (<25, 25 to 30, ≥30 kg/m^2^, or no data), fruits, nuts, and sugar-sweetened beverages consumption.

During a median follow-up of 3.9 years (interquartile range 3.4–4.9), we recorded 1437 new depression cases. Higher adherence to the SEAD was associated with lower risk of depression in the pooled sample [model 3 OR (95% CI) per 1-SD increment = 0.91 (0.86,0.96)]. The four countries showed a similar trend [Spain = 0.86 (0.75,0.99), Czechia = 0.86 (0.75,0.99), Poland = 0.97 (0.89,1.06), United Kingdom = 0.85 (0.75,0.97); *p* for interaction = 0.24] (Table 2, Fig. 1, Supplementary Fig. 2).

**Table 2:** Odds ratios (two-sided 95% confidence interval) for the association between adherence to the Southern European Atlantic diet and 3.9-year risk of depression.

	Southern European Atlantic diet			
	Lowest	Intermediate	Highest	Per 1-SD increment
Pooled sample				
Cases/n	449/3510	690/6217	298/3570	1437/13,297
Model 1^a^	Ref.	0.93 (0.82,1.06)	0.74 (0.63,0.87)***	0.91 (0.86,0.96)**
Model 2^b^	Ref.	0.93 (0.82,1.07)	0.74 (0.63,0.87)***	0.91 (0.86,0.96)**
Model 3^c^	Ref.	0.93 (0.81,1.06)	0.73 (0.62,0.86)***	0.91 (0.86,0.96)**
Spain				
Cases/n	70/582	110/1335	52/679	232/2596
Model 1^a^	Ref.	0.71 (0.51,0.97)*	0.68 (0.47,1.00)*	0.86 (0.75,0.99)*
Model 2^b^	Ref.	0.72 (0.52,0.99)*	0.69 (0.47,1.01)	0.87 (0.75,0.99)*
Model 3^c^	Ref.	0.71 (0.52,0.98)*	0.69 (0.47,1.01)	0.86 (0.75,0.99)*
Czechia				
Cases/n	75/886	120/1553	53/971	248/3410
Model 1^a^	Ref.	0.92 (0.68,1.24)	0.67 (0.46,0.97)*	0.86 (0.76,0.98)*
Model 2^b^	Ref.	0.93 (0.69,1.27)	0.68 (0.47,0.98)*	0.87 (0.76,0.99)*
Model 3^c^	Ref.	0.92 (0.68,1.26)	0.67 (0.46,0.97)*	0.86 (0.75,0.99)*
Poland				
Cases/n	233/1199	335/1645	133/826	701/3670
Model 1^a^	Ref.	1.05 (0.87,1.27)	0.83 (0.66,1.06)	0.97 (0.90,1.06)
Model 2^b^	Ref.	1.04 (0.86,1.26)	0.83 (0.65,1.05)	0.97 (0.89,1.06)
Model 3^c^	Ref.	1.04 (0.86,1.26)	0.82 (0.65,1.05)	0.97 (0.89,1.06)
UK				
Cases/n	71/843	125/1684	60/1094	256/3621
Model 1^a^	Ref.	0.88 (0.65,1.19)	0.64 (0.45,0.92)*	0.85 (0.75,0.97)*
Model 2^b^	Ref.	0.89 (0.65,1.21)	0.65 (0.45,0.93)*	0.86 (0.75,0.98)*
Model 3^c^	Ref.	0.88 (0.65,1.20)	0.64 (0.45,0.92)*	0.85 (0.75,0.97)*

*SD* standard deviation.

*p < 0.05; **p < 0.01; ***p < 0.001.

^a^Model 1: Logistic regression model adjusted for country (pooled sample), sex, age, educational level (primary or less, secondary, university, or no data), marital status (single, married/cohabiting, divorced/separated/widowed, or no data), and material deprivation (lower, intermediate, higher, or no data).

^b^Model 2: As Model 1 and additionally adjusted for smoking status (never, former, current, or no data), leisure-time physical activity (hours/week), and body mass index (<25, 25 to 30, ≥30 kg/m^2^, or no data).

^c^Model 3: As Model 2 and additionally adjusted for fruits, nuts, and sugar-sweetened beverages consumption.

Regarding the SEAD food groups, higher consumption of fresh fish, cod, red meat and pork products, legumes and vegetables, vegetable soup, potatoes, and whole-grain bread showed a tendency to lower risk of depression, contrary to that of dairy. However, these associations were non-significant and weaker than that of the SEAD as a whole. Only the consumption of small amounts of wine was associated with lower depression risk (Table 3, Supplementary Table 3).

**Table 3:** Odds ratios (two-sided 95% confidence interval) for the association between the Southern European Atlantic diet food groups and 3.9-year risk of depression in the pooled sample.

	Pooled sample			
	Cases/n	Model 1^a^	Model 2^b^	Model 3^c^
Fresh fish (excluding cod)				
≤Median	748/6649	Ref.	Ref.	Ref.
>Median	689/6645	0.93 (0.83,1.05)	0.93 (0.83,1.04)	0.96 (0.86,1.08)
Cod				
≤Median	822/7405	Ref.	Ref.	Ref.
>Median	615/5889	0.90 (0.80,1.01)	0.90 (0.80,1.01)	0.92 (0.81,1.03)
Red meat and pork products				
≤Median	728/6649	Ref.	Ref.	Ref.
>Median	709/6645	1.00 (0.89,1.11)	0.97 (0.86,1.09)	0.96 (0.86,1.08)
Dairy				
≤Median	704/6649	Ref.	Ref.	Ref.
>Median	733/6645	1.05 (0.94,1.18)	1.07 (0.95,1.20)	1.07 (0.95,1.20)
Legumes and vegetables				
≤Median	742/6650	Ref.	Ref.	Ref.
>Median	695/6647	0.94 (0.84,1.05)	0.94 (0.84,1.05)	0.96 (0.86,1.08)
Vegetable soup				
≤Median	817/7507	Ref.	Ref.	Ref.
>Median	620/5790	0.89 (0.79,1.01)	0.90 (0.80,1.01)	0.90 (0.80,1.02)
Potatoes				
≤Median	726/6649	Ref.	Ref.	Ref.
>Median	711/6645	0.96 (0.85,1.07)	0.94 (0.84,1.06)	0.95 (0.85,1.07)
Whole-grain bread				
≤Median	820/7396	Ref.	Ref.	Ref.
>Median	617/5898	0.90 (0.80,1.01)	0.91 (0.81,1.02)	0.91 (0.81,1.03)
Wine				
0 or >1 glass/day (women), 0 or >2 glasses/day (men)	915/6771	Ref.	Ref.	Ref.
≥0 to 1 glass/day (women), ≥0 to 2 glasses/day (men)	522/6523	0.84 (0.74,0.95)**	0.86 (0.76,0.97)*	0.86 (0.76,0.98)*

*p < 0.05; **p < 0.01.

^a^Model 1: Logistic regression model adjusted for country, sex, age, educational level (primary or less, secondary, university, or no data), marital status (single, married/cohabiting, divorced/separated/widowed, or no data), and material deprivation (lower, intermediate, higher, or no data).

^b^Model 2: As Model 1 and additionally adjusted for smoking status (never, former, current, or no data), leisure-time physical activity (hours/week), and body mass index (<25, 25 to 30, ≥30 kg/m^2^, or no data).

^c^Model 3: As Model 2 and additionally adjusted for all other SEAD food groups.

The protective association between the SEAD and risk of depression was of similar magnitude as that found for the AHEI [model 2 OR (95% CI) per 1-SD increment in the pooled sample = 0.93 (0.88,0.99)] and the MDS [0.94 (0.89,1.00)] (Supplementary Tables 4 and 5).

### Sensitivity analyses and interactions

Results from the main analyses held when calculating an alternate version of the SEAD considering total alcohol intake instead of wine consumption. The analyses were also robust to: (1) not adjusting for BMI; (2) excluding the subjects with diabetes or chronic lung disease; (3) accounting for baseline risk of probable depression; and (4) decreasing the number of depressive symptoms used to define depression (Supplementary Table 6).

We found no evidence that any of the sociodemographic, lifestyle, or dietary variables included in the models significantly modified the association between the SEAD and depression risk.

## Discussion

In this pooled analysis of cohorts from southern, central, eastern, and western European countries, higher adherence to the SEAD was associated with lower depression risk over a 3.9-year period. Results were rather consistent across countries. Most SEAD food groups showed a similar tendency, albeit non-significant. The association between the SEAD and risk of depression was of similar magnitude as that found for the AHEI and the MDS.

### Interpretation

Although a higher adherence to the SEAD has previously been associated with healthier gut microbiota, lower levels of several cardiovascular risk factors, and decreased risk of myocardial infarction and all-cause mortality [3, 13–21], no studies have examined the association of adherence to the SEAD with mood disorders. However, the effects of some of the SEAD food groups on depression have been studied extensively. A recent review of meta-analyses of prospective studies demonstrated a beneficial association of fish and vegetable consumption (moderate and low quality of evidence, respectively) and low/moderate alcohol intake (moderate quality of evidence), and a detrimental association of red and processed meat consumption (low quality of evidence). Moreover, higher adherence to the SEAD may be correlated with a nutrient pattern linked to lower risk of depression, specifically increased zinc, omega-3 fatty acid, and magnesium intake (moderate, low, and very low quality of evidence, respectively) [23].

There are credible explanations for these nutrients being potential predictors of depression. First, zinc participates in taste and smell perception, and its deficit induces anosmia. An important part of cerebral zinc is stored in the synaptic vesicles of glutaminergic neurons, and zinc deficiency may lead to depressive-like behaviour, sensitive to zinc supplementation [23, 40]. Second, omega-3 fatty acids have anti-inflammatory properties and play a role in the maintenance of cell membrane integrity and fluidity. Besides, these fatty acids act as natural ligands of peroxisome proliferator-activated receptor gamma, downregulating the neuronal inflammatory cascade in the pathophysiological process of depression [23]. Third, magnesium deficiency may lead to functional changes in the central nervous system (note that this micronutrient is a N-Methyl-D-aspartate receptor antagonist), upregulation of the stress response, and increased oxidative and inflammatory responses [41].

It is of note that, though we found a clear association between the SEAD and lower depression risk, none of its food groups -except for wine consumption- reached statistical significance. Also, the study associations were somewhat weaker when an alternate version of the SEAD (calculated with reverse scoring for red meat/pork products and potatoes, and without scoring wine consumption) was used. Any explanation for these findings must be conjectural. On one hand, dietary patterns can account for the small cumulative effects of food groups on chronic disease risk and for complex interactions between nutrients [8]. On the other hand, it is possible that some foods have a distinct health effect in younger and older subjects, as many of our study participants were over 60 years old. For instance, the high-quality protein content of meat may help delay sarcopenia, a common cause of physical disability and predictor of depression [42, 43]. Finally, the SEAD scoring for wine consumption does not account for the abstainer bias, the healthy drinker/survivor bias, or reverse causation [44], which may explain the beneficial association of this alcoholic beverage with depression in our study -note that 49% of the subjects who were given 0 points for wine consumption were never or former drinkers.

### Generalizability

Though there were no significant differences in study associations across countries, the relationship between the SEAD and risk of depression was consistently weaker in Poland (Table 2). Two hypotheses merit discussion. First, the consumption of the SEAD food groups differed by country, and equal SEAD scores could be obtained from substantially different combinations of food consumption. For instance, many participants from Poland did not drink wine, and this food group showed the strongest association with lower depression risk (Table 3, Supplementary Table 2). Second, those differences in study associations may reflect reverse causation. Despite excluding the subjects with cardiovascular disease or cancer history at baseline, 21% of our subjects suffered from diabetes or chronic lung disease. Since illness may lead individuals to change their dietary habits, this could have biased the study results [38]. It is therefore reassuring that excluding the subjects with any of these conditions strengthened the association between the SEAD and depression risk, and virtually made between-country variation disappear (p for country interaction = 0.73; Supplementary Table 6).

The generalizability of our findings to the targeted European countries may be limited, as response rates in the population-based cohorts included in this study were not always optimal [26–28, 32], and 22% of the subjects recruited at baseline were excluded from the analyses because of inadequate data. It should also be noted that most subjects from the Seniors-ENRICA and Whitehall-II cohorts were white/Caucasians (95.8%), and we lacked data on ethnicity in HAPIEE. This warrants caution when extrapolating our results to multi-ethnic/multiracial populations. Moreover, our sample comprised adults aged ≥45 years, and the presentation of psychological disorders may be different during adolescence and young adulthood as opposed to later in life. Nevertheless, we found no evidence that age significantly modified the study associations.

### Limitations

In previous validation studies, the correlation of Seniors-ENRICA’s diet history and Whitehall-II’s FFQ with seven 24-hour recalls was moderate (e.g., 0.76 and 0.34 for energy, 0.49 and 0.50 for fibre, and 0.69 and 0.81 for alcohol), though similar to that of other self-reported methods used to measure habitual diet [29, 33]. Despite no such data being available for HAPIEE’s FFQ, it was based on Whitehall-II’s, so similar results would have been expected [45]. A suitable strategy to overcome this limitation would have been to use repeated measurements of diet in the analyses, but we lacked such data -note that, in Seniors-ENRICA-2 and HAPIEE, no dietary data were collected in the follow-up waves. In any case, the inability to measure the true value of a dietary exposure would likely bias the study results towards the null [46].

Regarding the assessment of depression, some cases -particularly in their milder forms- were likely missed because of the rather long interval between data collection waves (3.9 years). In addition, the depression scales used differed across cohorts, and, in HAPIEE, different versions of the same scale were used at baseline and follow-up. Information on prescribed antidepressant medication was unavailable in HAPIEE, whereas self-reported diagnosis of depression was lacking in all cohorts but Seniors-ENRICA. Since depressive symptoms are often transient and may be linked to milder forms of depression, whereas major depressive disorder is of greater severity, the detection of depression and its accuracy likely differed from one cohort to another [22, 36]. Nonetheless, associations with the SEAD were rather consistent across cohorts.

Finally, there is potential for residual confounding, as many covariates were likely measured with some error, and some potential confounders could not be accounted for. First, cardiovascular disease history and cancer history were self-reported in the Seniors-ENRICA and HAPIEE cohorts. Second, grouping educational level, marital status, material deprivation, and tobacco smoking was necessary for data harmonization across cohorts, but led to loss of information. Third, we lacked data on leisure-time physical activity intensity and sedentary behaviours, and both may be associated with depression, independently of total physical activity time [47]. When taking these limitations together, the real range of uncertainty in odds ratios could be larger than that reflected in confidence intervals. It is reassuring, though, to see that minimally adjusted and fully adjusted models rendered similar results (even those accounting for habitual foods not included in the SEAD).

## Conclusions

In a pooled sample from four European countries, higher adherence to the SEAD was associated with lower 3.9-year risk of depression. Most food groups of the SEAD showed a similar tendency, albeit non-significant. These associations were of moderate magnitude, but consistent in main and sensitivity analyses. Given that diet is often measured with some error, can change over time and its effects on health could be cumulative and have long induction periods, more evidence from studies with repeated measurements of diet and longer-term follow-up would be desirable.

Together with the available studies on the SEAD, our findings suggest that the traditional dietary pattern of north-western Spain and northern Portugal could play a favourable role in both physical and mental health. Dietary recommendations for central, eastern, and western European countries could set their sights on the SEAD food groups that are culturally rooted in these regions (e.g., vegetable soups, dairy, and brown bread). The fact that the study associations were similar as those found for existing healthy dietary patterns, such as the AHEI and the MDS, implies that different diets could confer comparable benefits on depression risk.

### Supplementary information

Supplementary information

## Data Availability

The Seniors-ENRICA1, Seniors-ENRICA-2, HAPIEE, and Whitehall-II datasets used and/or analysed during the current study are available from the study authors on reasonable request.

## Code availability

The computer code used during the current study is available from the study authors on reasonable request.
