# Supplementary material for: The Southern European Atlantic diet and depression risk: a European multicohort study
Source: Mol Psychiatry. 2023 Jun 23;28(8):3475–83. doi: 10.1038/s41380-023-02125-9 (PMC10618086; doi:10.1038/s41380-023-02125-9)
Supplement: Supplementary file 1 — Supplementary information [file 41380_2023_2125_MOESM1_ESM.docx]

**TITLE PAGE**

**Title:** The Southern European Atlantic diet and depression risk: a European multicohort study

**Running title:** Southern European Atlantic diet and depression

**Authors’ names and affiliations:**

Adrián Carballo-Casla, PhD, ^a,b,c^ Denes Stefler, MD, PhD, ^b^ Rosario Ortolá, MD, PhD, ^a^ Yuntao Chen, PhD, ^b^ Anika Knuppel, PhD, ^d^ Milagros Ruiz, PhD, ^b,e^ Magdalena Kozela, PhD, ^f^ Ruzena Kubinova, MD, ^g^ Andrzej Pajak, MD, PhD, ^f^ Fernando Rodríguez-Artalejo, MD, PhD, ^a,h^ Eric J Brunner, PhD, ^b^ Martin Bobak, MD, PhD ^b^

^a^ Department of Preventive Medicine and Public Health, Universidad Autónoma de Madrid, Madrid, Spain. Center for Networked Biomedical Research in Epidemiology and Public Health (CIBERESP), Madrid, Spain.

^b^ Department of Epidemiology and Public Health, University College London, London, UK.

^c^ Aging Research Center, Department of Neurobiology, Care Sciences and Society, Karolinska Institutet & Stockholm University, Stockholm, Sweden.

^d^ Independent researcher.

^e^ School of Health and Social Care, University of Essex, Colchester, UK.

^f^ Department of Epidemiology and Population Studies, Jagiellonian University Medical College, Krakow, Poland.

^g^ National Institute of Public Health, Prague, Czech Republic.

^h^ IMDEA Research Institute on Food & Health Sciences. CEI UAM+CSIC, Madrid, Spain.

**Corresponding author:**

Adrián Carballo-Casla, PhD

Department of Preventive Medicine and Public Health, Universidad Autónoma de Madrid, Madrid, Spain. CIBER of Epidemiology and Public Health (CIBERESP), Madrid, Spain.

Department of Epidemiology and Public Health, University College London, London, UK.

Aging Research Center, Department of Neurobiology, Care Sciences and Society, Karolinska Institutet & Stockholm University, Stockholm, Sweden.

Telephone: (+34) 914972490. E-mail: adrian.carballo@uam.es or a.carballo@ucl.ac.uk

**Supplementary Appendix 1.** Categorization of the adherence to the Southern European Atlantic Diet.

Adherence categories (country-specific): Spain (Lowest, 0 to 2; Intermediate, 3 to 4; Highest, 5 to 8), Czechia (Lowest, 0 to 3; Intermediate, 4 to 5; Highest, 6 to 9), Poland (Lowest, 0 to 3; Intermediate, 4 to 5; Highest, 6 to 9), UK (Lowest, 0 to 3; Intermediate, 4 to 5; Highest, 6 to 9).

1-Standard Deviation increment (country-specific): Spain, 1.42; Czechia, 1.61; Poland, 1.59; UK, 1.59.

Restricted cubic spline knots (country-specific): Spain (knot 1, 2; knot 2, 4; knot 3, 5), Czechia (knot 1, 2; knot 2, 5; knot 3, 7), Poland (knot 1, 2; knot 2, 4; knot 3, 6), UK (knot 1, 3; knot 2, 5; knot 3, 7), reference (2).

**Supplementary Table 1.** Characteristics of the study individuals, by country.

|  | **Spain** | **Czechia** | **Poland** | **UK** |
| --- | --- | --- | --- | --- |
| n | 2596 | 3410 | 3670 | 3621 |
| Sex-Male (%) | 1406 (54.2) | 1631 (47.8) | 1902 (51.8) | 2714 (75.0)^*^ |
| Age (years) | 71.7 (5.09) | 57.8 (7.06) | 56.8 (6.90) | 60.9 (5.87)^*^ |
| Educational level (%) |  |  |  |  |
| Primary or less | 1357 (52.3) | 289 (8.48) | 267 (7.28) | 0 (0)^*^ |
| Secondary or vocational | 613 (23.6) | 2501 (73.3) | 2107 (57.4) | 2225 (61.4) |
| University | 625 (24.1) | 610 (17.9) | 1294 (35.3) | 1301 (35.9) |
| No data | 1 (0.04) | 10 (0.29) | 2 (0.05) | 95 (2.62) |
| Marital status (%) |  |  |  |  |
| Single | 177 (6.82) | 91 (2.67) | 192 (5.23) | 419 (11.6)^*^ |
| Married/cohabiting | 1865 (71.8) | 2659 (78.0) | 2983 (81.3) | 2845 (78.6) |
| Divorced/separated or widowed | 553 (21.3) | 651 (19.1) | 488 (13.3) | 338 (9.33) |
| No data | 1 (0.04) | 9 (0.26) | 7 (0.19) | 19 (0.52) |
| Material deprivation (%) |  |  |  |  |
| Lower | 1705 (65.7) | 1895 (55.6) | 2085 (56.8) | 1883 (52.0)^*^ |
| Intermediate | 804 (31.0) | 1394 (40.9) | 1289 (35.1) | 1648 (45.5) |
| Higher | 84 (3.24) | 102 (2.99) | 263 (7.17) | 25 (0.69) |
| No data | 3 (0.12) | 19 (0.56) | 33 (0.90) | 65 (1.80) |
| Tobacco smoking (%) |  |  |  |  |
| Never | 1344 (51.8) | 1615 (47.4) | 1541 (42.0) | 1778 (49.1)^*^ |
| Former | 1008 (38.8) | 935 (27.4) | 1011 (27.5) | 1588 (43.9) |
| Current | 244 (9.40) | 831 (24.4) | 1109 (30.2) | 235 (6.49) |
| No data | 0 (0) | 29 (0.85) | 9 (0.25) | 20 (0.55) |
| Body mass index (%) |  |  |  |  |
| <25 kg/m^2^ | 672 (25.9) | 961 (28.2) | 981 (26.7) | 1397 (38.6)^*^ |
| 25 to 30 kg/m^2^ | 1240 (47.8) | 1548 (45.4) | 1691 (46.1) | 1663 (45.9) |
| ≥30 kg/m^2^ | 682 (26.3) | 895 (26.2) | 996 (27.1) | 547 (15.1) |
| No data | 2 (0.08) | 6 (0.18) | 2 (0.05) | 14 (0.39) |
| Physical activity (hours/week) | 12.6 (9.25) | 18.2 (14.3) | 18.6 (12.8) | 12.0 (6.36)^*^ |
| Fruits (g/day) | 345 (183) | 418 (483) | 318 (252) | 335 (244)^*^ |
| Nuts (g/day) | 12.4 (17.5) | 4.14 (9.51) | 2.86 (9.12) | 4.86 (10.7)^*^ |
| Sugar-sweetened beverages (g/day) | 44.6 (111) | 189 (298) | 140 (162) | 174 (173)^*^ |

Values are numbers (%) or means (standard deviations).

^*^P-value <0.05 for differences in means (ANOVA) or proportions (Pearson’s chi-squared) across countries.

**Supplementary Table 2.** Median consumption of the Southern European Atlantic Diet food groups.

|  | **Spain** | | **Czechia** | | **Poland** | | **UK** | | **Pooled sample** | |
| --- | --- | --- | --- | --- | --- | --- | --- | --- | --- | --- |
|  | **Women** | **Men** | **Women** | **Women** | **Women** | **Men** | **Women** | **Men** | **Women** | **Men** |
| n | 1190 | 1406 | 1779 | 1631 | 1768 | 1902 | 907 | 2714 | 5644 | 7653 |
| **Fresh fish (excluding cod)** |  |  |  |  |  |  |  |  |  |  |
| Median (g/1000 kcal/day) | 29.0 | 27.2 | 4.75 | 4.08 | 6.53 | 6.85 | 6.57^*^ | 4.56^*^ | 7.51 | 6.21 |
| **Cod** |  |  |  |  |  |  |  |  |  |  |
| Median (g/1000 kcal/day) | 0 | 0 | 4.19 | 3.48 | 5.28 | 4.65 | 9.13^*^ | 8.43^*^ | 4.28 | 4.38 |
| **Red meat and pork products** |  |  |  |  |  |  |  |  |  |  |
| Median (g/1000 kcal/day) | 25.7 | 29.9 | 32.7 | 45.4 | 34.3 | 44.1 | 20.0^*^ | 23.8^*^ | 29.9 | 33.4 |
| **Dairy** |  |  |  |  |  |  |  |  |  |  |
| Median (g/1000 kcal/day) | 174 | 129 | 111 | 71.2 | 124 | 87.5 | 185^*^ | 162^*^ | 138 | 119 |
| **Legumes and vegetables** |  |  |  |  |  |  |  |  |  |  |
| Median (g/1000 kcal/day) | 129 | 124 | 130 | 94.8 | 120 | 101 | 144^*^ | 114^*^ | 129 | 109 |
| **Vegetable soup** |  |  |  |  |  |  |  |  |  |  |
| Median (g/1000 kcal/day) | 0 | 0 | 28.7 | 34.9 | 41.7 | 43.1 | 9.14^*^ | 7.79^*^ | 21.1 | 14.8 |
| **Potatoes** |  |  |  |  |  |  |  |  |  |  |
| Median (g/1000 kcal/day) | 19.7 | 21.0 | 44.6 | 43.7 | 41.9 | 43.8 | 53.4^*^ | 55.7^*^ | 39.6 | 43.9 |
| **Whole-grain bread** |  |  |  |  |  |  |  |  |  |  |
| Median (g/1000 kcal/day) | 0 | 0 | 19.2 | 18.6 | 17.0 | 5.99 | 19.9^*^ | 19.4^*^ | 14.9 | 11.7 |
| **Wine** |  |  |  |  |  |  |  |  |  |  |
| Median (glasses/day) | 0 | 0.48 | 0.05 | 0.05 | 0 | 0 | 0.36^*^ | 0.38^*^ | 0 | 0.09 |

^*^P-value <0.05 for sex-specific differences in means (ANOVA) across countries.

**Supplementary Table 3.** Odds Ratios (95% confidence interval) for the association between the Southern European Atlantic Diet food groups and 3.9-year risk of depression, by country.

|  | **Spain** | | **Czechia** | | **Poland** | | **UK** | |
| --- | --- | --- | --- | --- | --- | --- | --- | --- |
|  | **Cases/n** | **Model 3 ^a^** | **Cases/n** | **Model 3 ^a^** | **Cases/n** | **Model 3 ^a^** | **Cases/n** | **Model 3 ^a^** |
| **Fresh fish (excluding cod)** |  |  |  |  |  |  |  |  |
| ≤ Median | 126/1297 | Ref. | 134/1706 | Ref. | 361/1835 | Ref. | 127/1811 | Ref. |
| > Median | 106/1296 | 0.86 (0.65,1.13) | 114/1704 | 0.92 (0.71,1.21) | 340/1835 | 0.98 (0.83,1.17) | 129/1810 | 1.06 (0.82,1.37) |
| **Cod** |  |  |  |  |  |  |  |  |
| ≤ Median | 186/2053 | Ref. | 146/1706 | Ref. | 355/1835 | Ref. | 135/1811 | Ref. |
| > Median | 46/540 | 0.97 (0.69,1.37) | 102/1704 | 0.72 (0.55,0.94)* | 346/1835 | 1.01 (0.85,1.20) | 121/1810 | 0.88 (0.68,1.14) |
| **Red meat and pork products** |  |  |  |  |  |  |  |  |
| ≤ Median | 121/1297 | Ref. | 117/1706 | Ref. | 358/1835 | Ref. | 132/1811 | Ref. |
| > Median | 111/1296 | 0.91 (0.69,1.20) | 131/1704 | 1.13 (0.87,1.48) | 343/1835 | 0.94 (0.79,1.12) | 124/1810 | 0.91 (0.71,1.19) |
| **Dairy** |  |  |  |  |  |  |  |  |
| ≤ Median | 105/1297 | Ref. | 135/1706 | Ref. | 336/1835 | Ref. | 128/1811 | Ref. |
| > Median | 127/1296 | 1.25 (0.95,1.65) | 113/1704 | 0.84 (0.65,1.09) | 365/1835 | 1.14 (0.96,1.35) | 128/1810 | 1.00 (0.77,1.29) |
| **Legumes and vegetables** |  |  |  |  |  |  |  |  |
| ≤ Median | 131/1298 | Ref. | 127/1706 | Ref. | 350/1835 | Ref. | 134/1811 | Ref. |
| > Median | 101/1298 | 0.78 (0.59,1.03) | 121/1704 | 0.98 (0.76,1.28) | 351/1835 | 1.05 (0.88,1.24) | 122/1810 | 0.94 (0.72,1.21) |
| **Vegetable soup** |  |  |  |  |  |  |  |  |
| ≤ Median | 190/2155 | Ref. | 136/1706 | Ref. | 353/1835 | Ref. | 138/1811 | Ref. |
| > Median | 42/441 | 1.08 (0.76,1.55) | 112/1704 | 0.81 (0.62,1.05) | 348/1835 | 0.93 (0.78,1.10) | 118/1810 | 0.86 (0.67,1.12) |
| **Potatoes** |  |  |  |  |  |  |  |  |
| ≤ Median | 125/1297 | Ref. | 126/1706 | Ref. | 347/1835 | Ref. | 128/1811 | Ref. |
| > Median | 107/1296 | 0.82 (0.62,1.07) | 122/1704 | 0.93 (0.71,1.21) | 354/1835 | 1.00 (0.84,1.18) | 128/1810 | 1.01 (0.78,1.31) |
| **Whole-grain bread** |  |  |  |  |  |  |  |  |
| ≤ Median | 184/2044 | Ref. | 128/1706 | Ref. | 369/1835 | Ref. | 139/1811 | Ref. |
| > Median | 48/549 | 0.89 (0.64,1.26) | 120/1704 | 0.94 (0.73,1.23) | 332/1835 | 0.93 (0.79,1.11) | 117/1810 | 0.85 (0.66,1.10) |
| **Wine** |  |  |  |  |  |  |  |  |
| 0 or >1 glass/day (women), 0 or >2 glasses/day (men) | 142/1268 | Ref. | 117/1505 | Ref. | 547/2785 | Ref. | 109/1213 | Ref. |
| ≥ 0 to 1 glass/day (women), ≥ 0 to 2 glasses/day (men) | 90/1325 | 0.74 (0.56,0.98)* | 131/1905 | 1.03 (0.79,1.34) | 154/885 | 0.96 (0.78,1.18) | 147/2408 | 0.70 (0.54,0.91)** |

*p<0.05. **p<0.01.

^a^ Model 3: Logistic regression model adjusted as Model 3 in Table 3: sex, age, educational level (primary or less, secondary, university, or no data), marital status (single, married/cohabiting, divorced/separated/widowed, or no data), material deprivation (lower, intermediate, higher, or no data), smoking status (never, former, current, or no data), leisure-time physical activity (hours/week), body mass index (<25, 25 to 30, ≥30 kg/m^2^, or no data), and all other SEAD food groups.

**Supplementary Table 4.** Odds Ratios (95% confidence interval) for the association between adherence to the Alternate Healthy Eating Index and 3.9-year risk of depression.

|  | **Alternate Healthy Eating Index ^a^** | | | |
| --- | --- | --- | --- | --- |
|  | **Lowest** | **Intermediate** | **Highest** | **Per 1-SD increment** |
| **Pooled sample** |  |  |  |  |
| Cases/n | 516/4434 | 454/4432 | 467/4431 | 1437/13297 |
| Model 1 ^b^ | Ref. | 0.82 (0.72,0.95)** | 0.85 (0.74,0.98)* | 0.92 (0.87,0.97)** |
| Model 2 ^c^ | Ref. | 0.84 (0.73,0.96)* | 0.88 (0.77,1.01) | 0.93 (0.88,0.99)* |
| **Spain** |  |  |  |  |
| Cases/n | 89/866 | 80/865 | 63/865 | 232/2596 |
| Model 1 ^b^ | Ref. | 0.90 (0.65,1.24) | 0.75 (0.53,1.05) | 0.81 (0.71,0.93)** |
| Model 2 ^c^ | Ref. | 0.91 (0.66,1.26) | 0.78 (0.55,1.09) | 0.82 (0.72,0.94)** |
| **Czechia** |  |  |  |  |
| Cases/n | 94/1137 | 77/1137 | 77/1136 | 248/3410 |
| Model 1 ^b^ | Ref. | 0.74 (0.54,1.01) | 0.72 (0.52,0.98)* | 0.88 (0.77,1.01) |
| Model 2 ^c^ | Ref. | 0.76 (0.55,1.04) | 0.76 (0.55,1.04) | 0.90 (0.79,1.03) |
| **Poland** |  |  |  |  |
| Cases/n | 242/1224 | 216/1223 | 243/1223 | 701/3670 |
| Model 1 ^b^ | Ref. | 0.82 (0.67,1.01) | 0.94 (0.77,1.16) | 0.96 (0.88,1.04) |
| Model 2 ^c^ | Ref. | 0.83 (0.68,1.03) | 0.97 (0.79,1.19) | 0.97 (0.89,1.06) |
| **UK** |  |  |  |  |
| Cases/n | 91/1207 | 81/1207 | 84/1207 | 256/3621 |
| Model 1 ^b^ | Ref. | 0.87 (0.63,1.18) | 0.88 (0.65,1.21) | 0.97 (0.85,1.10) |
| Model 2 ^c^ | Ref. | 0.88 (0.64,1.21) | 0.90 (0.66,1.23) | 0.98 (0.86,1.11) |

*p<0.05. SD = Standard Deviation.

^a^ Adherence categories (country-specific): Spain (Lowest, 20 to 59.6; Intermediate, 59.6 to 68.1; Highest, 68.1 to 93.3; 1-SD increment, 9.83), Czechia (Lowest, 15.8 to 41.6; Intermediate, 41.6 to 50.4; Highest, 50.4 to 79.1; 1-SD increment, 9.75), Poland (Lowest, 18.8 to 38.4; Intermediate, 38.4 to 45.6; Highest, 45.6 to 82.5; 1-SD increment, 8.32), UK (Lowest, 20.1 to 45.9; Intermediate, 45.9 to 53.8; Highest, 53.8 to 77.7; 1-SD increment, 8.91).

^b^ Model 1: Logistic regression model adjusted for country (pooled sample), sex, age, educational level (primary or less, secondary, university, or no data), marital status (single, married/cohabiting, divorced/separated/widowed, or no data), and material deprivation (lower, intermediate, higher, or no data).

^c^ Model 2: As Model 1 and additionally adjusted for smoking status (never, former, current, or no data), leisure-time physical activity (hours/week), and body mass index (<25, 25 to 30, ≥30 kg/m^2^, or no data).

**Supplementary Table 5.** Odds Ratios (95% confidence interval) for the association between adherence to the Mediterranean Diet Score and 3.9-year risk of depression.

|  | **Mediterranean Diet Score ^a^** | | | |
| --- | --- | --- | --- | --- |
|  | **Lowest** | **Intermediate** | **Highest** | **Per 1-SD increment** |
| **Pooled sample** |  |  |  |  |
| Cases/n | 519/4261 | 413/4412 | 505/4621 | 1437/13294 |
| Model 1 ^b^ | Ref. | 0.84 (0.73,0.97)* | 0.90 (0.79,1.03) | 0.94 (0.89,0.99)* |
| Model 2 ^c^ | Ref. | 0.85 (0.73,0.97)* | 0.91 (0.80,1.04) | 0.94 (0.89,1.00)* |
| **Spain** |  |  |  |  |
| Cases/n | 91/793 | 91/1133 | 50/667 | 232/2593 |
| Model 1 ^b^ | Ref. | 0.72 (0.53,0.98)* | 0.67 (0.47,0.97)* | 0.84 (0.74,0.97)* |
| Model 2 ^c^ | Ref. | 0.73 (0.53,0.99)* | 0.68 (0.47,0.99)* | 0.85 (0.74,0.98)* |
| **Czechia** |  |  |  |  |
| Cases/n | 85/1083 | 59/846 | 104/1481 | 248/3410 |
| Model 1 ^b^ | Ref. | 0.90 (0.63,1.27) | 0.93 (0.69,1.26) | 0.93 (0.82,1.07) |
| Model 2 ^c^ | Ref. | 0.88 (0.62,1.25) | 0.95 (0.70,1.28) | 0.94 (0.82,1.08) |
| **Poland** |  |  |  |  |
| Cases/n | 260/1295 | 163/885 | 278/1490 | 701/3670 |
| Model 1 ^b^ | Ref. | 0.88 (0.70,1.10) | 0.94 (0.77,1.14) | 0.98 (0.90,1.07) |
| Model 2 ^c^ | Ref. | 0.90 (0.72,1.12) | 0.94 (0.78,1.14) | 0.99 (0.91,1.07) |
| **UK** |  |  |  |  |
| Cases/n | 83/1090 | 100/1548 | 73/983 | 256/3621 |
| Model 1 ^b^ | Ref. | 0.84 (0.62,1.13) | 1.00 (0.72,1.39) | 0.92 (0.81,1.05) |
| Model 2 ^c^ | Ref. | 0.84 (0.62,1.14) | 1.01 (0.73,1.41) | 0.93 (0.82,1.06) |

*p<0.05. **p<0.01. SD = Standard Deviation.

^a^ Adherence categories (country-specific): Spain (Lowest, 0 to 3; Intermediate, 4 to 5; Highest, 6 to 9; 1-SD increment, 1.66), Czechia (Lowest, 0 to 3; Intermediate, 4; Highest, 5 to 9; 1-SD increment, 1.61), Poland (Lowest, 0 to 3; Intermediate, 4; Highest, 5 to 9; 1-SD increment, 1.53), UK (Lowest, 0 to 3; Intermediate, 4 to 5; Highest, 6 to 9; 1-SD increment, 1.73).

^b^ Model 1: Logistic regression model adjusted for country (pooled sample), sex, age, educational level (primary or less, secondary, university, or no data), marital status (single, married/cohabiting, divorced/separated/widowed, or no data), and material deprivation (lower, intermediate, higher, or no data).

^c^ Model 2: As Model 1 and additionally adjusted for smoking status (never, former, current, or no data), leisure-time physical activity (hours/week), and body mass index (<25, 25 to 30, ≥30 kg/m^2^, or no data).

**Supplementary Table 6.** Sensitivity analyses. Odds Ratios (95% confidence interval) for the association between adherence to the Southern European Atlantic Diet (per 1-standard deviation increment) and 3.9-year risk of depression.

|  | **Cases/n** | **Model 1 ^a^** | **Model 2 ^b^** | **Model 3 ^c^** |
| --- | --- | --- | --- | --- |
| **Pooled sample** |  |  |  |  |
| SEAD optimised for potential public health interventions ^d^ | 1437/13297 | 0.95 (0.90,1.01) | 0.96 (0.91,1.02) | 0.96 (0.91,1.02) |
| SEAD considering total alcohol intake ^e^ | 1437/13297 | 0.92 (0.86,0.97)** | 0.92 (0.86,0.97)** | 0.91 (0.86,0.97)** |
| Not adjusting for BMI | 1437/13297 | 0.91 (0.86,0.96)** | 0.91 (0.86,0.97)** | 0.91 (0.86,0.96)** |
| Excluding the subjects with diabetes or chronic lung disease | 1057/10507 | 0.89 (0.84,0.96)*** | 0.90 (0.84,0.96)** | 0.90 (0.84,0.96)** |
| Accounting for baseline risk of probable depression | 1437/13297 | 0.91 (0.86,0.97)** | 0.92 (0.86,0.97)** | 0.91 (0.86,0.97)** |
| Decreasing the number of depressive symptoms used to define depression ^f^ | 1661/11343 | 0.94 (0.89,0.99)* | 0.94 (0.89,0.99)* | 0.94 (0.89,0.99)* |
| **Spain** |  |  |  |  |
| SEAD optimised for potential public health interventions ^d^ | 232/2596 | 1.00 (0.87,1.15) | 1.01 (0.88,1.16) | 1.02 (0.88,1.17) |
| SEAD considering total alcohol intake ^e^ | 232/2596 | 0.91 (0.79,1.04) | 0.92 (0.80,1.05) | 0.91 (0.80,1.05) |
| Not adjusting for BMI | 232/2596 | 0.86 (0.75,0.99)* | 0.87 (0.75,0.99)* | 0.86 (0.75,0.99)* |
| Excluding the subjects with diabetes or chronic lung disease | 151/1931 | 0.85 (0.72,1.01) | 0.86 (0.72,1.02) | 0.86 (0.72,1.02) |
| Accounting for baseline risk of probable depression | 232/2596 | 0.88 (0.76,1.01) | 0.88 (0.77,1.01) | 0.88 (0.76,1.01) |
| Decreasing the number of depressive symptoms used to define depression ^f^ | 327/2418 | 0.88 (0.78,0.99)* | 0.88 (0.78,0.99)* | 0.88 (0.78,0.99)* |
| **Czechia** |  |  |  |  |
| SEAD optimised for potential public health interventions ^d^ | 248/3410 | 0.85 (0.74,0.96)* | 0.86 (0.76,0.98)* | 0.86 (0.76,0.98)* |
| SEAD considering total alcohol intake ^e^ | 248/3410 | 0.86 (0.75,0.98)* | 0.86 (0.76,0.99)* | 0.86 (0.75,0.98)* |
| Not adjusting for BMI | 248/3410 | 0.86 (0.76,0.98)* | 0.87 (0.77,1.00)* | 0.87 (0.76,0.99)* |
| Excluding the subjects with diabetes or chronic lung disease | 173/2600 | 0.85 (0.73,0.99)* | 0.86 (0.74,1.01) | 0.86 (0.73,1.01) |
| Accounting for baseline risk of probable depression | 248/3410 | 0.87 (0.76,0.99)* | 0.88 (0.77,1.00)* | 0.87 (0.76,1.00)* |
| Decreasing the number of depressive symptoms used to define depression ^f^ | 277/2734 | 0.92 (0.81,1.04) | 0.92 (0.82,1.05) | 0.93 (0.82,1.06) |
| **Poland** |  |  |  |  |
| SEAD optimised for potential public health interventions ^d^ | 701/3670 | 0.99 (0.91,1.08) | 1.00 (0.92,1.09) | 1.00 (0.92,1.09) |
| SEAD considering total alcohol intake ^e^ | 701/3670 | 0.96 (0.88,1.05) | 0.96 (0.88,1.04) | 0.95 (0.88,1.04) |
| Not adjusting for BMI | 701/3670 | 0.97 (0.90,1.06) | 0.98 (0.90,1.06) | 0.97 (0.90,1.06) |
| Excluding the subjects with diabetes or chronic lung disease | 540/3025 | 0.94 (0.85,1.03) | 0.94 (0.85,1.03) | 0.94 (0.85,1.03) |
| Accounting for baseline risk of probable depression | 701/3670 | 0.97 (0.89,1.05) | 0.97 (0.89,1.05) | 0.96 (0.88,1.05) |
| Decreasing the number of depressive symptoms used to define depression ^f^ | 705/2955 | 1.00 (0.92,1.09) | 1.00 (0.92,1.09) | 1.00 (0.92,1.09) |
| **UK** |  |  |  |  |
| SEAD optimised for potential public health interventions ^d^ | 256/3621 | 0.93 (0.82,1.05) | 0.93 (0.82,1.06) | 0.94 (0.82,1.06) |
| SEAD considering total alcohol intake ^e^ | 256/3621 | 0.87 (0.77,0.99)* | 0.87 (0.77,0.99)* | 0.87 (0.76,0.99)* |
| Not adjusting for BMI | 256/3621 | 0.85 (0.75,0.97)* | 0.86 (0.75,0.97)* | 0.85 (0.75,0.97)* |
| Excluding the subjects with diabetes or chronic lung disease | 193/2951 | 0.87 (0.75,1.01) | 0.87 (0.75,1.01) | 0.87 (0.75,1.01) |
| Accounting for baseline risk of probable depression | 256/3621 | 0.87 (0.77,0.99)* | 0.88 (0.77,1.00)* | 0.87 (0.77,1.00)* |
| Decreasing the number of depressive symptoms used to define depression ^f^ | 352/3236 | 0.90 (0.80,1.01) | 0.90 (0.81,1.01) | 0.90 (0.81,1.01) |

*p<0.05. **p<0.01. ***p<0.001.

^a^ Model 1: Logistic regression model adjusted for country (pooled sample), sex, age, educational level (primary or less, secondary, university, or no data), marital status (single, married/cohabiting, divorced/separated/widowed, or no data), and material deprivation (lower, intermediate, higher, or no data).

^b^ Model 2: As Model 1 and additionally adjusted for smoking status (never, former, current, or no data), leisure-time physical activity (hours/week), and body mass index (<25, 25 to 30, ≥30 kg/m^2^, or no data).

^c^ Model 3: As Model 2 and additionally adjusted for fruits, nuts, and sugar-sweetened beverages consumption.

^d^ Reverse scoring the consumption of red meat/pork products and potatoes, and not scoring wine consumption. Adjusting for wine consumption.

^e^ Men who had >0 and ≤20 g/day of alcohol, and women who had >0 and ≤10 g/day of alcohol were given 1 point, whereas no points were given for >20 g/day in men, >10 g/day in women, or 0 g/day.

^f^ ≥2 points (Geriatric Depression Scale); ≥3 points (Center for Epidemiological Studies Depression 10); ≥12 points (Center for Epidemiological Studies Depression 20).

**Supplementary Figure 1.** Participants’ flow chart.

POOLED SAMPLE (total=32,344)

Participants in the Seniors-ENRICA-1 study at wave 1 (n=2,519)

Participants in the Seniors-ENRICA-2 study (n=3,273)

Participants in the HAPIEE study (n=19,585)

Participants in the Whitehall-II study at phase 5 (n=6,967)

INELIGIBLE (total=11,929) ^a^

Prevalent depression at baseline (n=7,149)

Cardiovascular disease or cancer history at baseline (n=6,800)

- Cardiovascular disease history (n=5,442)
- Cancer history (n=1,743)

Participants free of depression, cardiovascular disease, and cancer at baseline

n=20,415

EXCLUDED (total=7,118) ^b^

No data on diet (total=1,501)

- No data on diet at baseline (n=1,501)

No data on depression (total=6,194)

- No data on depression at baseline (n=626)
- No data on depression at follow-up (n=5,862)

**Analytical sample**

**n=13,297**

^a^ Note that some participants suffered from multiple conditions.

^b^ Note that some participants lacked data in more than one variable.

**Supplementary Figure 2.** Odds Ratios (95% confidence interval) for the association between adherence to the Southern European Atlantic Diet and 3.9-year risk of depression, by country.

**
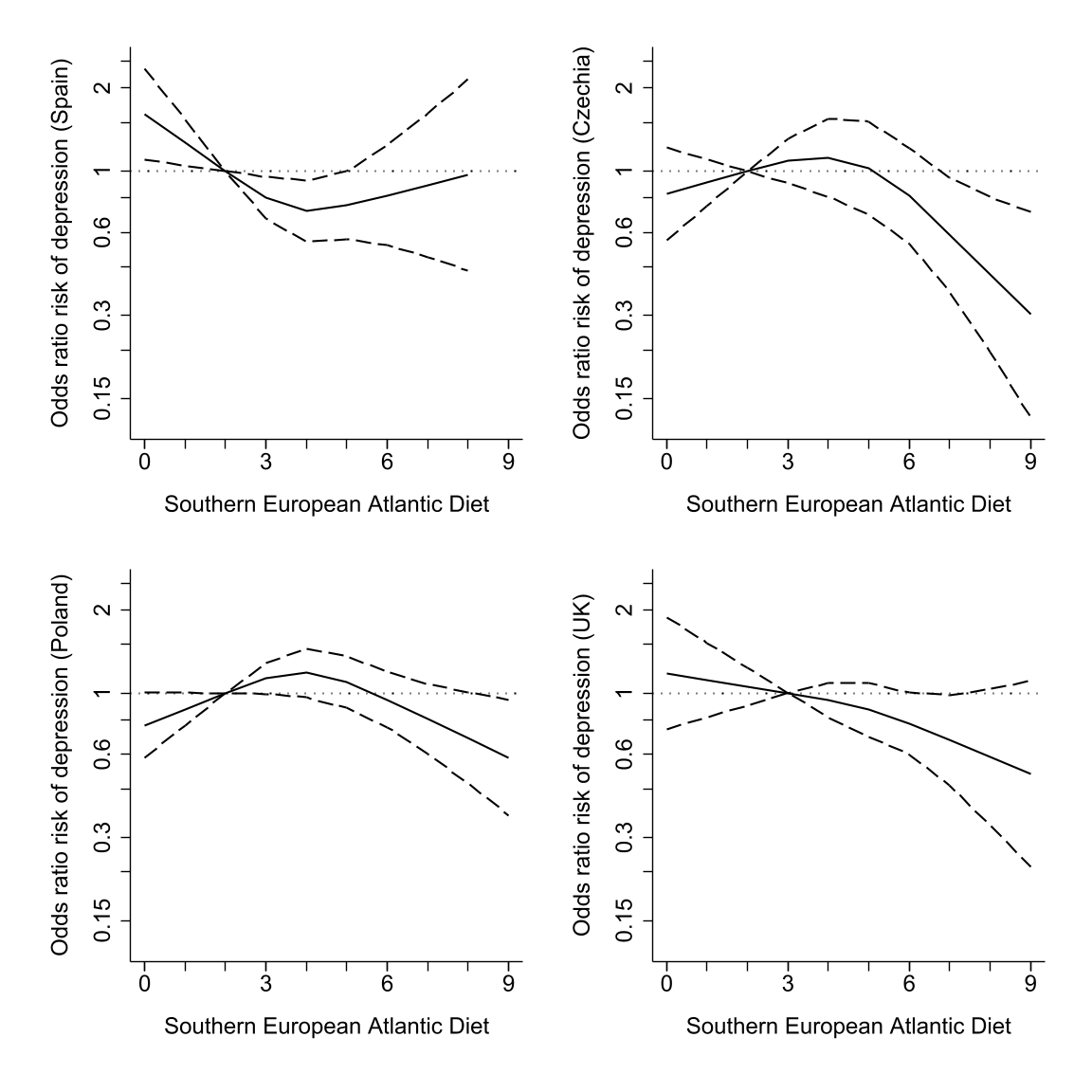
**

Logistic regression model adjusted as Model 3 in Table 2: sex, age, educational level (primary or less, secondary, university, or no data), marital status (single, married/cohabiting, divorced/separated/widowed, or no data), material deprivation (lower, intermediate, higher, or no data), smoking status (never, former, current, or no data), leisure-time physical activity (hours/week), body mass index (<25, 25 to 30, ≥30 kg/m^2^, or no data), fruits, nuts, and sugar-sweetened beverages consumption.

Restricted cubic spline knots (country-specific): Spain (knot 1, 2; knot 2, 4; knot 3, 5), Czechia (knot 1, 2; knot 2, 5; knot 3, 7), Poland (knot 1, 2; knot 2, 4; knot 3, 6), UK (knot 1, 3; knot 2, 5; knot 3, 7), reference (2).
